# The use of buprenorphine/naloxone to treat borderline personality disorder: a case report

**DOI:** 10.1186/s40479-022-00181-1

**Published:** 2022-03-15

**Authors:** Brenna Hansen, Katelyn M. Inch, Brenna A. Kaschor

**Affiliations:** 1 Department of Emergency Medicine, University of Alberta, T6G 2E1 Edmonton, Alberta Canada; 2 Department of Health Sciences, Western University, N6A 3K7 London, Ontario Canada; 3 Prisma Health Care Collaborative, N5Z 3L2 London, Ontario Canada; 4 Department of Family Medicine, Schulich School of Medicine and Dentistry, Western University, N6G 2M1 London, Ontario Canada; 5 Mental Health and Addictions Program, London Health Sciences Center, N6A5W9 London, Ontario Canada

**Keywords:** Borderline Personality Disorder, Opioids, Pharmacotherapy, Psychopharmacology Substance-Related and Addictive Disorders

## Abstract

**Background:**

Using traditional pharmacotherapy to treat Borderline Personality Disorder (BPD) such as mood stabilizers and second-generation antipsychotics has a lack of supporting evidence. Buprenorphine/Naloxone (BUP/N), a combination medication consisting of a partial opioid agonist, and a full opioid antagonist, is an effective treatment for opioid use disorder. It has also been found effective for treatment-resistant mood disorders. Previous studies suggest a relationship between BPD and endogenous opioids, therefore our case report investigates the effect of BUP/N on a patient diagnosed with BPD.

**Case presentation:**

A 26-year-old female diagnosed with BPD, having recurrent visits to the emergency department (ED) for self-harm/suicidality was treated with BUP/N. Usage of crisis services, ED visits, and hospital admissions were tracked from 15 months prior to BUP/N to 15 months after using BUP/N. Since starting BUP/N, the length and frequency of mental health-related hospital admissions decreased drastically, as did the number of times that she reached out to community crisis services. Since the dosing adjustment to 6 mg in Oct 2020, there have been no calls to the community crisis lines.

**Conclusions:**

We suggest pharmacological treatment targeting BPD as a disorder of distress tolerance and self-soothing mediated by the opioid system is an effective individual healing attempt. An important note is that this patient did not use opioids prior to BUP/N and had never been diagnosed with an opioid use disorder. However, she exhausted multiple other pharmacologic therapies and was open to trying whatever was available to improve her quality of life.

## Background

Borderline Personality Disorder (BPD) is a complex diagnosis, with little agreement among providers regarding effective treatment. Patients access health services frequently, have difficulty maintaining stable relationships, and show poor distress tolerance. Traditional pharmacotherapy focuses on mood/anxiety/emotional dysregulation symptoms using mood stabilizers and second-generation antipsychotics - despite limited supporting evidence. [1] Studies investigating the physiologic basis have found that BPD patients have more ***µ***-opioid receptors in their brains than controls, specifically in the orbitofrontal cortex bilaterally, the caudate nucleus bilaterally, extending into the nucleus accumbens on the right, the left nucleus accumbens, and the left amygdala, [2] suggesting a paucity of endogenous opioids, compensated by an increased number of ***µ***-opioid receptors. [2] Distress vocalizations have been well studied in various animal models [3] and are either quelled or potentiated by low-dose opioid agonists or antagonists, respectively. The similarities between distress vocalizations and behaviours seen with BPD suggests a common mechanism, both rooted in the body’s intrinsic need for attachment. [4].

Buprenorphine/Naloxone (BUP/N), a partial opioid agonist [5] combined with a full antagonist with limited oral bioavailability, is well known as an effective treatment for Opioid Use Disorder. This case report investigates ***µ***-opioid receptor agonism of the opioid system in BPD patients, and therefore preparations such as naltrexone and nalmefene, which are both opioid receptor antagonists, were not investigated in this context. Given this relationship between BPD and endogenous opioids, we propose BUP/N as a highly effective and neurobiologically rational treatment, demonstrated by the following case:

## Case presentation

A 26-year-old patient with a history of BPD had a 12-year history of emergency department (ED) presentations and admissions for suicidality/self-harm, despite years of psychiatric support and trials of multiple medications from multiple drug classes. She is supported on social assistance, does not work, lives alone in an apartment with her emotional support dog and has no family interactions. Prior to starting BUP/N, she had been on multiple psychiatric medications throughout her long course of treatment. These included antipsychotics, stimulants, SSRIs, SNRIs, NDRIs, and benzodiazepines. At one point in her treatment, she had tried every other class of psychotropic medications. She had no physical illnesses.

From 2017 to March 2020, she had 18 ED visits with 9 admissions. In March 2020 she started on 2 mg of BUP/N, increased to 4 mg in April, and to 6 mg in October.

Her use of hospital and community-based crisis services was assessed before and after BUP/N. She accessed community crisis services substantially less (41 vs. 12 events) in 15 months prior to BUP/N, compared to the following 15 months with BUP/N. She has not accessed these services at all since her dose was increased to 6 mg in October. Data for 39 months before and 13 months after BUP/N initiation for access to hospital-based crisis services also shows a substantial reduction. Total average time in hospital, including ED and inpatient, decreased from 216.4 h/visit to 13.7 h/visit. Admissions/year decreased from 2.8/year to 1.8/year, and average length of admission decreased from 401.2 h to 63 h. During the process of preparing and submitting the manuscript, the patient did discontinue the medication for a short time. She ended up in hospital with suicidal thoughts and emotional dysregulation as before, which resolved again when she started back on the medication. The patient had been engaged in various forms of therapy before and during the use of BUP/N including Dialectical Behaviour Therapy (DBT) and Cognitive Behavioural Therapy (CBT). There was no material change in the therapy she received before compared with after starting BUP/N. No scales of function were completed, however the patient continued to engage with her nurse case manager throughout. Reading those clinical notes, the patient is described as going about her day and caring for herself in the usual manners. The study took place during the first part of the COVID-19 pandemic, and as such there were limited activities to do in general outside the house.

Most importantly throughout her journey, she describes a substantial increase in quality of life and sense of wellbeing. The patient objectively felt better taking BUP/N, and therefore did not want to discontinue use.

## Discussion and Conclusion

We suggest that pharmacological treatment targeting BPD as a disorder of distress tolerance and self-soothing mediated by the opioid system, rather than a mood/anxiety disorder mediated by monoamine neurotransmitters, is an effective individual healing attempt. Although we realize that severe BPD is associated with greater monoamine oxidase-A total distribution volume (MAO-A V_T_) as well, and therefore could be a target for pharmacotherapy, [6] MAO inhibitors can lead to lethal consequences due to medication and food contraindications or death due to intentional overdose. [7] Borderline personality disorder has been described as interpersonal difficulties, a series of behaviours that leads to difficulties with self-regulation and regulation within the context of relationships. [8] If we look at these behaviours, the description is very similar to the behaviours surrounding attachment cry/separation anxiety in infants and children. There is an inability to self-soothe, resulting in pervasive reaching out and seeking for another to do this. As a secure attachment pattern develops in childhood, there is an increased ability to explore and self-regulate. [9] The neurobiology of attachment cry is well known and is mediated by the opioid system, specifically the ***µ*** opioid system. [3] We hypothesize that this is because traditional opioids will activate both ***µ*** and **κ**, resulting in the attenuation of the attachment cry, but also in the dysphoria associated with **κ** activation. Buprenorphine is unique in that it provides agonism at ***µ*** and antagonism at **κ**. Therefore, we would expect an attenuation of the attachment cry, the drive for connection, without a concomitant increase in dysphoria and dissociation, which is exactly what we have described in this case.

We propose that individuals with BPD are less able to independently soothe the PANIC/GRIEF primary process affective system. [3] Low levels of ***µ***-receptor activation combined with **κ**-receptor blockade from Buprenorphine, combined with oxytocin-inducing effects of a positive therapeutic relationship, can attenuate the PANIC/GRIEF system, and individuals no longer need to reach out for co-regulation. [3] This may have contributed to the beneficial effects seen in this patient. The implications of this treatment for both the individual’s sense of agency and healthcare system utilization cannot be understated.

## Data Availability

The data that support the findings of this study are not publicly available due to their containing information that could compromise the privacy of the research participant, but are available from the corresponding author BAK upon reasonable request.

## Abbreviations

BPD: Borderline Personality Disorder
BUP/N: Buprenorphine/Naloxone
CBT: Cognitive Behavioural Therapy
DBT: Dialectical Behaviour Therapy
ED: Emergency Department
MAO: Monoamine Oxidase
MAO-A V_T_: Monoamine Oxidase-A total distribution volume
